# Betulinic Acid Inhibits the Stemness of Gastric Cancer Cells by Regulating the GRP78-TGF-β1 Signaling Pathway and Macrophage Polarization

**DOI:** 10.3390/molecules28041725

**Published:** 2023-02-11

**Authors:** Jen-Lung Chen, Yun-Shen Tai, Hsin-Yi Tsai, Chia-Yuan Hsieh, Chun-Lin Chen, Chung-Jung Liu, Deng-Chyang Wu, Yaw-Bin Huang, Ming-Wei Lin

**Affiliations:** 1 Department of Surgery, E-Da Hospital, Kaohsiung 82445, Taiwan; 2 Department of Surgery, China Medical University, An-Nan Hospital, Tainan 70965, Taiwan; 3 School of Pharmacy, Kaohsiung Medical University, Kaohsiung 80708, Taiwan; 4 Department of Medical Research, E-Da Hospital/E-Da Cancer Hospital, Kaohsiung 82445, Taiwan; 5 Department of Biological Science, National Sun Yat-sen University, Kaohsiung 80424, Taiwan; 6 Division of Gastroenterology, Department of Internal Medicine, Kaohsiung Medical University Hospital, Kaohsiung 80708, Taiwan; 7 Regenerative Medicine and Cell Therapy Research Center, Kaohsiung Medical University, Kaohsiung 80708, Taiwan; 8 Center for Cancer Research, Kaohsiung Medical University, Kaohsiung 80708, Taiwan; 9 Department of Nursing, College of Medicine, I-Shou University, Kaohsiung 82445, Taiwan

**Keywords:** betulinic acid, stemness, gastric cancer, GRP78, TGF-β, macrophage polarization

## Abstract

Cancer stemness is the process by which cancer cells acquire chemoresistance and self-renewal in the tumor microenvironment. Glucose-regulated protein 78 (GRP78) is a biomarker for gastric cancer and is involved in cancer stemness. By inducing cancer stemness in various types of cancer, the polarization of macrophages into tumor-associated macrophages (TAMs) controls tumor progression. Betulinic acid (BA) is a bioactive natural compound with anticancer properties. However, whether GRP78 regulates TAM-mediated cancer stemness in the tumor microenvironment and whether BA inhibits GRP78-mediated cancer stemness in gastric cancer remain unknown. In this study, we investigated the role of GRP78 in gastric cancer stemness in a tumor microenvironment regulated by BA. The results indicated that BA inhibited not only GRP78-mediated stemness-related protein expression and GRP78-TGF-β-mediated macrophage polarization into TAMs, but also TAM-mediated cancer stemness. Therefore, BA is a promising candidate for clinical application in combination-chemotherapy targeting cancer stemness.

## 1. Introduction

The therapeutic efficacy of gastric cancer treatment has remained limited because cancer cells acquire stemness through self-renewal [1]. Previous studies have indicated that cancer stemness plays an essential role in chemoresistance and tumor recurrence [2]. Gastric cancer stem cells express high levels of the surface marker CD44 and the stemness-related transcription factor OCT4 [3]. These CD44^+^ gastric cancer cells are invasive and resistant to chemotherapy [4]. CD44 interaction with OCT4 signaling plays a critical role in the acquisition of cancer stem-cell properties, including chemoresistance, in cancer cells [5]. Glucose-regulated protein 78 (GRP78) is an endoplasmic reticulum chaperon that produces an adaptive response in order to promote cell survival under stress [6]. This protein has been identified as a human gastric-cancer biomarker and has been linked to gastric cancer stem cell-like characteristics, stemness-related protein expression, and cancer-associated fibroblast activation in the tumor microenvironment [7]. However, the role in GRP78-mediated cancer stemness undertaken by other cells, particularly macrophages, in the tumor microenvironment remains under investigation.

Generally, M1/M2 macrophage polarization regulates the progression of tumors in various cancers [8,9]. M1 macrophages expressing inducible nitric oxide synthase (iNOS) are regarded as antitumor macrophages, whereas M2-polarized macrophages expressing arginase 1 (Arg1) modulate cancer-related activities, including cancer stemness [10]. To promote cancer cell proliferation, metastasis, and chemoresistance, cancer cells induce macrophage M2 phenotype polarization in the tumor microenvironment [8,9]. In addition, the transforming growth factor β (TGF-β) secreted by cancer cells or the tumor microenvironment promotes the differentiation of nonactivated macrophages into a tumor-associated macrophage (TAM) phenotype while suppressing proinflammatory M1 phenotypes [11]. This positive feedback, generated by TGF-β loops, drives the polarization of macrophages toward the protumor TAM phenotype. Additionally, the modeling of macrophage polarization influences the efficacy of cancer treatment [12]. Therefore, inhibition of TGF-β-induced macrophage polarization is essential for TAM-induced chemoresistance related to cancer stemness.

Betulinic acid (BA) is a bioactive pentacyclic triterpenoid present in white birch. Although some studies have reported that BA exhibits anti-inflammatory and anticancer properties and can be used to treat neglected tropical diseases [13,14,15], only a few studies have investigated the antiproliferative and migration effects of BA against gastric cancer cells [16,17]. In addition, the ability of BA to regulate the tumor microenvironment by modulating TAM polarization and gastric cancer stemness remains unclear.

Cancer stemness is a potential therapeutic strategy in cancer treatment. Both cancer stemness targeting and combined chemotherapy may assist in inhibiting tumor growth and preventing cancer recurrence [18]. In this study, we examined the stemness of human gastric cancer cells at molecular level and the regulation of GRP78/TGF-β1-mediated TAM polarization and interleukin-6 (IL-6) secretion by BA. We then investigated the inhibitory effect of IL-6 reduction on gastric-cancer cell stemness.

## 2. Results

### 2.1. BA Inhibits the Expression of GRP78, TGF-β1, and Stemness Markers in Human Gastric Cancer Cells

**Figure 1:**
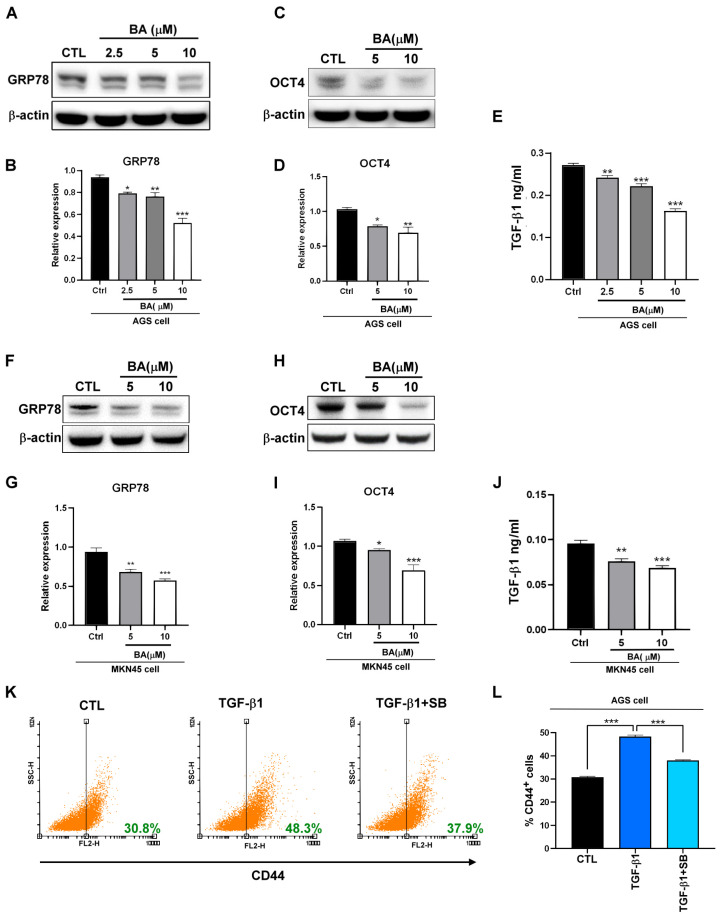
(**A**,**B**) GRP78 expression and quantification by Western blotting. Human gastric cancer AGS cells were treated with 2.5, 5, and 10 μM BA for 72 h, which inhibited the expression of GRP78. (**C**) TGF-β1 levels after treatment of AGS cells with 2.5, 5, and 10 μM BA in a conditioned medium. (**D**,**E**) OCT4 expression and quantification by Western blotting after treatment of AGS cells with 5 and 10 μM BA for 72 h. (**F**,**G**) GRP78 expression and quantification by Western blotting after treatment of MKN45 cells with 5 and 10 μM BA for 72 h. (**H**,**I**) OCT4 expression and quantification by Western blotting after treatment of MKN45 cells with 5 and 10 μM BA for 72 h. (**J**) TGF-β1 levels in media conditioned after treatment of MKN45 cells with BA. (**K**,**L**) CD44^+^ AGS cells analyzed using flow cytometry after treatment with 20 ng/mL TGF-β1 and 10 μM SB431542 (TGF-β receptor inhibitor) for 48 h. Data are presented as mean ± standard error of the mean (SEM) for four independent experiments. Significance was calculated using one-way ANOVA analysis: * *p* < 0.05, ** *p* < 0.01, *** *p* < 0.005.

GRP78 is involved in the expression of TGF-β and the stemness of gastric cancer cells [7]. However, whether BA inhibits GRP78 to regulate stemness and TGF-β remains unclear. In this study, we confirmed that BA downregulates the expression of GRP78 in human gastric-cancer AGS cells (Figure 1A,B). To determine whether BA inhibits gastric cancer-cell stemness through a GRP78-TGF-β-mediated pathway, we evaluated TGF-β1 and the expression of the stemness-related transcription factor OCT4 in AGS cells after BA treatment. The results indicated that BA inhibited TGF-β/Smad2/3 signaling, TGF-β1 secretion, and OCT4 expression in a dose-dependent manner (Figure 1C–E). Similar results were also observed in MKN45, another human gastric cancer cell line (Figure 1F–J). OCT4 is a key regulator that maintains the pluripotency and self-renewal of cancer stem cells (CSCs) [18]. Knockdown of the cell surface stemness marker CD44 suppresses stem cell-like properties by downregulating OCT4 [19,20]. In addition, TGF-β1 promotes the growth of CD44^+^ populations in AGS, and the TGF-β inhibitor SB431542 reverses the TGF-β1-induced increase in CD44^+^ cells (Figure 1K,L). These findings indicate that GRP78 and TGF-β are involved in the expression of stemness-related markers in BA-treated human gastric cancer AGS cells (Supplementary Material).

### 2.2. BA Contributes to the GRP78-TGF-β1-Mediated Inhibition of Gastric Cancer Stemness

**Figure 2:**
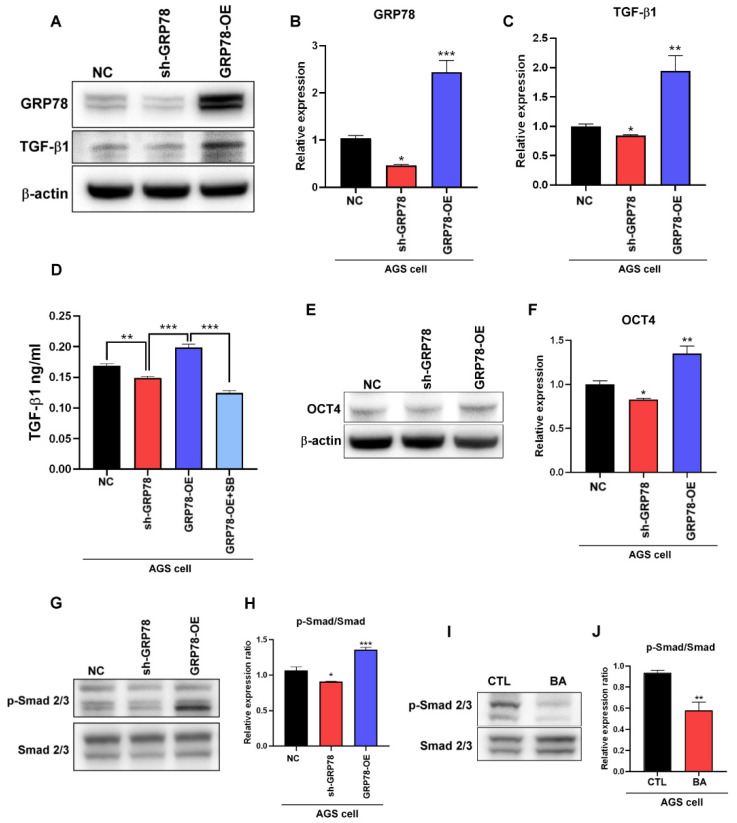
(**A**–**C**) GRP78 and TGF-β1 expression and quantification by Western blotting for NC, sh-GRP78, and GRP78-OE AGS cells. (**D**) TGF-β1 levels in conditioned media with NC, sh-GRP78, and GRP78-OE AGS cells. (**E**,**F**) OCT4 expression and quantification by Western blotting for NC, sh-GRP78, and GRP78-OE AGS cells. (**G**,**H**) p-Smad2/3 and Smad2/3 protein expression and quantification by Western blotting for NC, sh-GRP78, and GRP78-OE AGS cells. Data are presented as mean ± SEM for three independent experiments. Significance was calculated by one-way ANOVA analysis: * *p* < 0.05, ** *p* < 0.01, *** *p* < 0.005. (**I**,**J**) p-Smad2/3 and Smad2/3 protein expression and quantification by Western blotting after treatment of AGS cells with 5 μM BA for 72 h. Data are presented as mean ± SEM for three independent experiments. Two-tailed Student’s *t*-test: * *p* < 0.05 and ** *p* < 0.01.

In colon cancer, GRP78 overexpression promotes epithelial–mesenchymal transition through autocrine TGF-β/Smad2/3 signaling [21]. According to previous studies, TGF-β1 promotes the expression of the pluripotent transcription factor OCT4 and cancer stemness characteristics [22,23]. In this study, to determine whether GRP78 regulates the expression of the TGF-β1-mediated stemness marker OCT4 in human gastric cancer, we evaluated the expression of TGF-β1 and OCT4 in AGS cells with GRP78 overexpression and knockdown by using Western blotting and enzyme-linked immunosorbent assay (ELISA). The results indicated that the GRP78 level in AGS cells regulated the expression of TGF-β1 and OCT4 (Figure 2A–F). In addition, TGF-β1 activated the Smad2/3 signaling pathway and regulated the transcription process through both Smad2 and Smad3 [24]. Smad2/3 downregulation also enhanced the suppression of OCT4 [25,26]. These findings indicate that GRP78 and BA regulate the activation of Smad2/3 (Figure 2G–J), suggesting that BA may regulate the expression of the human gastric-cancer stemness marker OCT4 through the GRP78-TGF-β1-Smad2/3 signaling pathway.

### 2.3. TGF-β1 Regulates Macrophages TAM-Type Polarization

**Figure 3:**
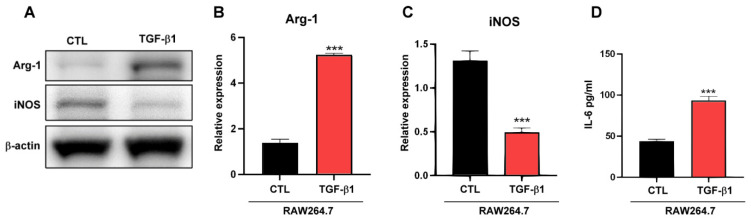
(**A**–**C**) Arg1 and iNOS protein expression and quantification by Western blotting after treatment of RAW 264.7 cells with 20 ng/mL TGF-β1 for 48 h. (**D**) IL-6 levels in conditioned media after treatment of RAW 264.7 cells with TGF-β1. Data are presented as mean ± SEM for four independent experiments. Two-tailed Student’s *t*-test: *** *p* < 0.005.

Generally, TAMs facilitate tumor progression [27], and TGF-β, which is secreted by cancer cells, promotes the differentiation of macrophages into M2-like TAMs [28]. As depicted in Figure 1A–C, BA regulated the expression of TGF-β1 in gastric cancer cells, confirming that TGF-β1 plays a role in macrophage polarization. As indicated in Figure 3A–D, TGF-β1 induced macrophages to express the M2-type marker Arg1 and downregulated the expression of the M1-type marker iNOS. In addition, IL-6 derived from TAMs increased the CSC population [29], and TGF-β1 regulated the expression of IL-6 in macrophages (Figure 3D).

### 2.4. Gastric Cancer Cells Overexpressing GRP78 Induce M2-Type Macrophages through the TGF-β1 Signaling Pathway

**Figure 4:**
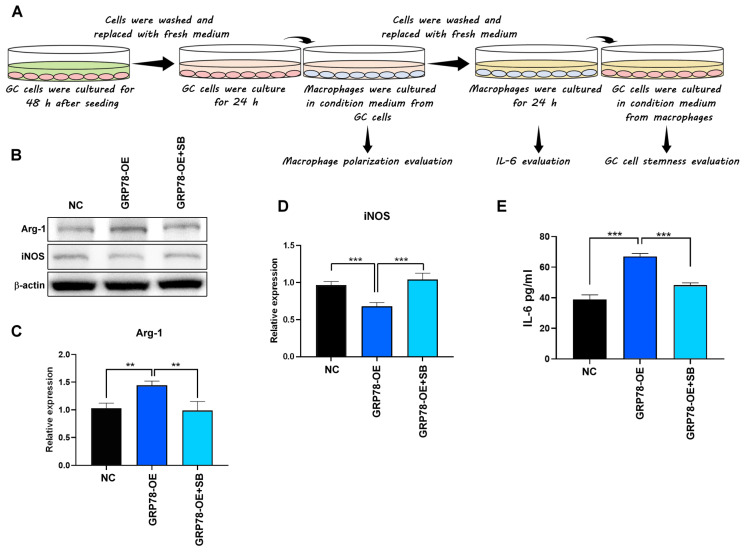
(**A**) Macrophage polarization assays. (**B**–**D**) Arg1 and iNOS protein expression and quantification by Western blotting after treatment with an AGS-conditioned medium for 48 h. (**E**) Macrophages were cultured in conditioned medium from AGS cells (NC or GRP78-OE or GRP78-OE+SB431542) for 48 h. The medium was then replaced with a fresh culture medium. After 24 h incubation, the medium was collected for IL-6 assay. The IL-6 level in the medium was evaluated using the IL-6 ELISA kit. Data are presented as mean ± SEM for three independent experiments. Significance was calculated using one-way ANOVA analysis: ** *p* < 0.01 and *** *p* < 0.005.

To determine whether GRP78-expressing gastric cancer cells regulate macrophage polarization through the TGF-β1 signaling pathway, macrophages were treated with either a conditioned medium containing GRP78-overexpressing gastric cancer cells or a conditioned medium containing the TGF-β1 receptor inhibitor SB431542. NC, GRP78-OE, and GRP78-OE AGS cells treated with SB431542 were then cultured for 48 h. As shown in Figure 4A, which depicts macrophage polarization assays, the cells were washed, placed in a fresh medium containing 1% FBS, and cultured for 24 h to generate a cancer cell-conditioned medium (shown in pink). RAW 264.7 cells were then incubated for 48 h in the cancer-conditioned medium. The medium was subsequently removed, and the macrophages were cultured in a fresh medium for 24 h to generate a macrophage-conditioned medium (shown in yellow). The IL-6 concentration in the macrophage-conditioned medium was then analyzed using ELISA. After 48 h of incubation in the macrophage-conditioned medium, the AGS cells were analyzed using flow cytometry. The results indicated that the conditioned medium with GRP78-overexpressing gastric cancer cells promoted the expression of the TAM/M2 marker Arg1 and suppressed the expression of the M1 marker iNOS in macrophages. In addition, SB431542, a TGF-β1 receptor inhibitor, reversed the TAM-type promotion effects, indicating that the GRP78-overexpressing gastric cancer cells promoted TAM-type macrophages through the TGF-β1 signaling pathway (Figure 4B–D). Generally, IL-6 derived from TAMs is a potent inducer that enriches CSC populations [29]. In this study, IL-6 levels were increased in macrophages treated with a conditioned medium containing GRP78-OE and decreased in GRP78-OE containing SB431542 (Figure 4E), indicating that gastric cancer induced macrophage IL-6 elevation through the GRP78-TGF-β1 pathway.

### 2.5. IL-6 Promotes Gastric Cancer Stemness

**Figure 5:**
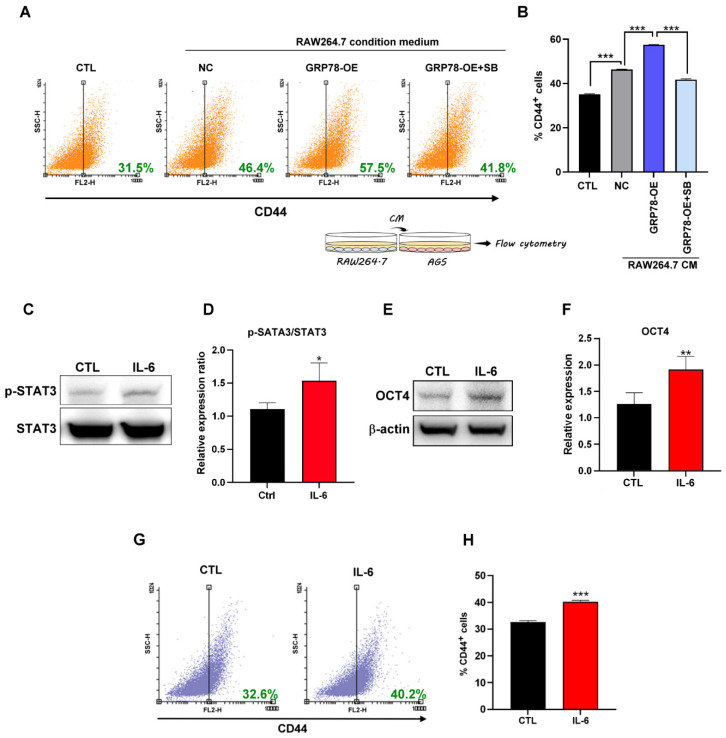
(**A**,**B**) Surface marker CD44 analyzed using flow cytometry in AGS after macrophage-conditioned medium treatment for 48 h. Data are presented as mean ± SEM for four independent experiments. Significance was calculated by one-way ANOVA analysis: *** *p* < 0.005. (**C**,**D**) p-STAT3 and STAT3 protein expression and quantification by Western blotting after treatment of AGS with 20 ng/mL IL-6 for 48 h. (**E**,**F**) OCT4 quantification by Western blotting after treatment of AGS with 20 ng/mL IL-6 for 48 h. (**G**,**H**) Surface marker CD44 analyzed using flow cytometry in AGS after treatment with 20 ng/mL IL-6 for 48 h. Data are presented as mean ± SEM for three independent experiments. Two-tailed Student’s *t*-test: * *p* < 0.05, ** *p* < 0.01 and *** *p* < 0.005.

The results confirmed that GRP78-overexpressing gastric cancer cells induced macrophage IL-6 expression and that the conditioned medium containing macrophages (Figure 4A) enriched the CD44^+^ gastric CSC population through the TGF-β1 signaling pathway (Figure 5A,B). IL-6 is a TAM-produced cytokine that is involved in tumor progression and metastasis through the STAT3 signaling pathway and is responsible for the prosurvival adaptation of tumor cells to hypoxia [29,30,31]. In this study, macrophage-derived IL-6 was regulated by the GRP78-mediated secretion of TGF-β1 in gastric cancer cells. As shown in Figure 5C–H, IL-6 activated the STAT3 signaling pathway and induced cancer stemness by upregulating the expression of OCT4 and CD44 in gastric cancer cells.

## 3. Discussion

Chemotherapy is the primary treatment method for solid cancers. However, M2-like TAMs secrete cytokines that promote tumor aggressiveness and cancer stemness through epithelial–mesenchymal transition [32]. TAM-derived IL-6 induces tumorigenic and invasive effects and promotes breast, colorectal, and hepatocellular cancer or gastric tumor chemoresistance through the STAT3 signaling pathway [29,33,34,35]. In non-small-cell lung cancer, IL-10 derived from M2 macrophages promotes cancer stemness through the JAK1/STAT1/NF-κB/Notch1 pathway [36]. In addition, TAM-induced IGF promotes thyroid cancer stemness and metastasis by activating the PI3K/AKT/mTOR pathway [37], and TGF-β induces M2-like macrophage polarization [11]. Therefore, the suppression of macrophage polarization into M2-like TAMs in the tumor microenvironment may inhibit TAM-derived IL-6-induced cancer stemness.

**Figure 6:**
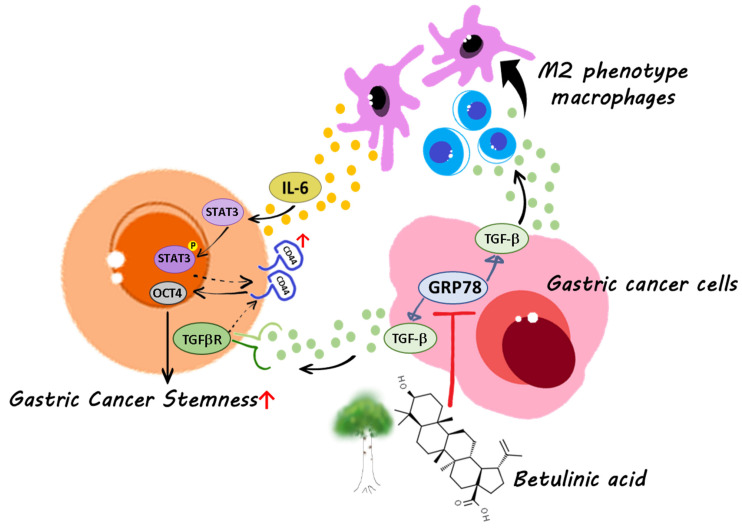
Scheme of gastric cancer stemness inhibition by BA through GRP78/TGF-β1 signaling and macrophage polarization.

Small natural compounds, including BA, are potential candidates for combined therapy. Overall, this research has demonstrated the importance of GRP78-mediated cancer stemness in gastric cancer [7]. Generally, the stemness of gastric cancer can be identified by analyzing the expression of cell surface markers (CD44) or stemness-related transcriptional factors (OCT4). In addition, pluripotency proteins are co-expressed by CD44^+^ cells, and OCT4 participates in self-renewal and chemoresistance and may represent the stemness of gastric cancer [38,39,40]. In accordance with our findings, we suggest that BA may regulate the microenvironment of gastric tumors to eliminate cancer stemness by downregulating the stemness-related transcription factor OCT4. The results revealed that GRP78 regulated the expression of TGF-β1 and TGF-β1-mediated stemness in human gastric cancer cells and that TGF-β1 derived from gastric cancer cells promoted macrophage polarization and IL-6 secretion. In addition, IL-6 stimulated gastric cancer stemness by activating the STAT3 signaling pathway and upregulating the stemness-related marker OCT4. Moreover, BA dose-dependently inhibited the GRP78-mediated secretion of TGF-β, thereby suppressing macrophage polarization, IL-6 secretion, and IL-6-mediated gastric cancer stemness (Figure 6). In a previous study, TGF-β signaling was reported to involve a positive feedback loop in the regulation of triple-negative breast cancer and macrophage-mediated cancer stemness and progression [41]. The results also indicated that BA-induced downregulation of TGF-β through the GRP78-mediated pathway may inhibit the positive feedback loop between gastric cancer cells and macrophage-mediated cancer stemness and tumor progression.

In addition to inducing GRP78/TGF-β-mediated IL-6 expression, BA may directly inhibit the secretion of IL-6 by modulating NF-κB in mononuclear cells [42]. BA may target both cancer cells and macrophages to suppress cancer stemness in the tumor microenvironment.

## 4. Materials and Methods

### 4.1. Cell Culture and Reagent

An AGS human gastric cancer cell line (CRL-1739) was purchased from the American Type Culture Collection (ATCC, Manassas, VA, USA). AGS cells were maintained in an RPMI 1640 (Gibco, Waltham, MA, USA) medium supplemented with 10% fetal bovine serum (FBS; Gibco, Waltham, MA, USA) under 5% CO_2_ at 37 °C. AGS/NC (shLacZ, clone ID: TRCN231722), AGS/GRP78-OE (GRP78-Bip-pLAS2w cloning vector), and AGS/sh-GRP78 (shHSPA5, clone ID: TRCN218611) were purchased from the National RNAi Core Facility (RNA Technology Platform and Gene Manipulation Core, Academia Sinica, Taipei, Taiwan). Transfected AGS cells were cultured in an RPMI 1640 medium (Gibco) containing 10% FBS and 1.5 μg/mL puromycin under 5% CO_2_ at 37 °C. A RAW 264.7 monocyte/macrophage-like cell line (TIB-71) was purchased from the ATCC and cultured in Dulbecco’s modified Eagle’s medium (Gibco) supplemented with 10% FBS under 5% CO_2_ at 37 °C. Stock solutions of BA (Sigma-Aldrich, St. Louis, MO, USA) and SB431542 (Sigma-Aldrich) in dimethyl sulfoxide (Sigma-Aldrich) were prepared and dissolved in the culture medium before treatment. Recombinant human TGF-β1 (GFH39-5; Cell Guidance Systems, Cambridge, UK) and recombinant human IL-6 (CYT-1150; ProSpec, East Brunswick, NJ, USA) were prepared and dissolved in the culture medium before treatment.

### 4.2. Western Blot Analysis

Human gastric cancer cells were washed with phosphate-buffered saline (PBS). Total protein samples were then extracted, and protein concentrations were measured using the Bradford protein assay (Bio-Rad, Hercules, CA, USA). Equal quantities of total proteins were separated using Bolt Bis-Tris Plus 4–12% sodium dodecyl sulfate-polyacrylamide gel electrophoresis (Thermo Fisher Scientific, New York, NY, USA) and transferred onto polyvinylidene fluoride membranes. After the membranes were washed with PBS and Tween 20, they were then blocked with a blocking buffer (Bio-Rad) for 30 min at room temperature and incubated with primary antibodies GRP78 (1:1000; Cell Signaling Technology, Danvers, MA, USA), OCT4 (1:1000; Abcam, Cambridge, UK), β-actin (1:5000; Cell Signaling Technology), TGF-β1 (1:1000; Abcam), Smad2/3 (1:1000; Cell Signaling Technology), phospho-Smad2/3 (p-Smad2/3, 1:1000; Cell Signaling Technology), Arg1 (1:1000; Proteintech, Martinsried, Planegg, Germany), iNOS (1:1000; Abcam), STAT3 (1:1000; Cell Signaling Technology), and phospho-STAT3 (p-STAT3, 1:1000; Cell Signaling Technology) at 4 °C. Finally, the membranes were incubated with secondary antibodies at room temperature for 1 h and analyzed using an electrochemiluminescence detection system.

### 4.3. Measurement of TGF-β1 Level

Conditioned medium was obtained from AGS treated with BA or from AGS/NC, AGS/sh-GRP78, or AGS/GRP78-OE treated with SB431542. The TGF-β1 level was then determined using a TGF-β1 enzyme-linked immunosorbent assay (ELISA) kit (Abcam) in accordance with the manufacturer’s instructions.

### 4.4. Measurement of IL-6 Level

Conditioned medium was obtained from RAW 264.7 cells treated with TGF-β1 or from AGS/NC or AGS/GRP78-OE treated with SB431542. The IL-6 level was then determined using an IL-6 ELISA kit (Abcam) in accordance with the manufacturer’s instructions.

### 4.5. Stimulation of Cells with Conditioned Media

To generate conditioned media from AGS/NC, AGS/GRP78-OE, or AGS/GRP78-OE treated with SB431542 (GRP78-OE+SB), gastric cancer cells (2 × 10^6^) were seeded in 10 cm dishes and cultured in a medium containing 1% FBS for 2 days before being washed. A fresh nonconditioned medium was then added, and the gastric cancer cells were cultured for 24 h. Subsequently, the medium conditioned by gastric cancer cells was filtered, evaluated using ELISA, and used with RAW 264.1 cells. To evaluate the polarization of macrophages, RAW 264.7 cells were incubated in a tumor-conditioned medium for 2 days and then washed. A fresh nonconditioned medium was then added, and the RAW 264.7 cells were cultured for 24 h. Subsequently, the cells were analyzed using Western blotting, and the medium conditioned by RAW 264.7 was filtered, evaluated using ELISA, and used with AGS cells. To evaluate the stemness of gastric cancer cells, AGS cells were incubated in a macrophage-conditioned medium for 2 days, and cancer stemness was analyzed using Western blotting and flow cytometry 2 days thereafter.

### 4.6. Flow Cytometry Analysis

After treatment, the cells were washed with cold PBS and stained for 45 min with a surface marker antibody. After being stained, the cells were washed twice with cold PBS before analysis. Flow cytometry was then used to evaluate the expression of CSC markers (CD44; BD Biosciences, San Jose, CA, USA) on human gastric cancer cells.

### 4.7. Statistical Analysis

All data were analyzed using GraphPad Prism version 8 (GraphPad Software, version 8, San Diego, CA, USA). The results are presented as mean ± SEM. Significance calculated from Student’s *t*-test or one-way ANOVA analysis was set at *p* < 0.05.

## 5. Conclusions

Targeting cancer stemness in gastric tumors by using natural compounds may be an effective treatment option for patients with gastric cancer. BA inhibits gastric cancer stemness by regulating GRP78/TGF-β1 in gastric cancer cells, TAM polarization, and macrophage-derived IL-6 signaling in the tumor microenvironment. Therefore, BA is regarded as a promising reagent in clinical combination therapy.

## Data Availability

All data sets generated or analyzed in this study were included in the published article. Detailed data sets supporting the current study are available from the corresponding author upon request. This study did not generate new codes.

## Supplementary Materials

The following supporting information can be downloaded at: https://www.mdpi.com/article/10.3390/molecules28041725/s1, Figure S1: The human gastric cancer cells, AGS or MKN45 were treated BA from 2.5–10 μM for 48 h. The cell viability was evaluated by CCK-8 assay. The results revealed that BA inhibited human gastric cancer growth at 10 μM. Data are presented as mean ± SEM for four independent experiments. Significance was calculated by one-way ANOVA analysis: ** *p* < 0.01; Figure S2: The human gastric cancer cells, AGS or MKN45 were treated BA from 2.5–10 μM for 48 h. The active caspase 3/7 was evaluated by using FITC Active Caspase-3 Apoptosis Kit. The results demonstrated that BA induced caspase 3/7 activation in human gastric cancer cells. Data are presented as mean ± SEM for four independent experiments. Significance was calculated by one way ANOVA analysis: *** *p* < 0.005; Figure S3: Surface marker CD44 histogram analyzed by flow cytometry after treatment with 20 ng/mL TGF-β1 and 10 μM SB431542 (TGF-β receptor inhibitor) for 48 h. Data are presented as mean ± SEM for four independent experiments. Significance was calculated by oneway ANOVA analysis: *** *p* < 0.005; Figure S4: Surface marker CD44 histogram analyzed by flow cytometry in AGS after conditioned medium treatment for 48 h. Data are presented as mean ± SEM for four independent experiments. Significance was calculated by one-way ANOVA analysis: *** *p* < 0.005; Figure S5: Surface marker CD44 histogram analyzed by flow cytometry in AGS after treatment with 20 ng/mL IL-6 for 48 h. Data are presented as mean ± SEM for four independent experiments. Significance was calculated by one-way ANOVA analysis: *** *p* < 0.005.; Figure S6: GRP78 expression by Western blotting analysis. Human gastric cancer MKN45 cells were treated with 2.5, 5, and 10 μM BA for 48 h. BA at 2.5 μM did not inhibit GRP78 within 48 h in MKN45. However, BA inhibited GRP78 from the dosage of 2.5 to 10 μM in AGS; Cell viability analysis; Cell viability analysis; Western blot analysis.

## Sample Availability

A sample of the compound, betulinic acid, is available from the authors.
