# Supplementary material for: Betulinic Acid Inhibits the Stemness of Gastric Cancer Cells by Regulating the GRP78-TGF-β1 Signaling Pathway and Macrophage Polarization
Source: Molecules. 2023 Feb 11;28(4):1725. doi: 10.3390/molecules28041725 (PMC9964887; doi:10.3390/molecules28041725)
Supplement: Supplementary file 1 [file molecules-28-01725-s001.zip › molecules-2090295-supplementary.pdf]

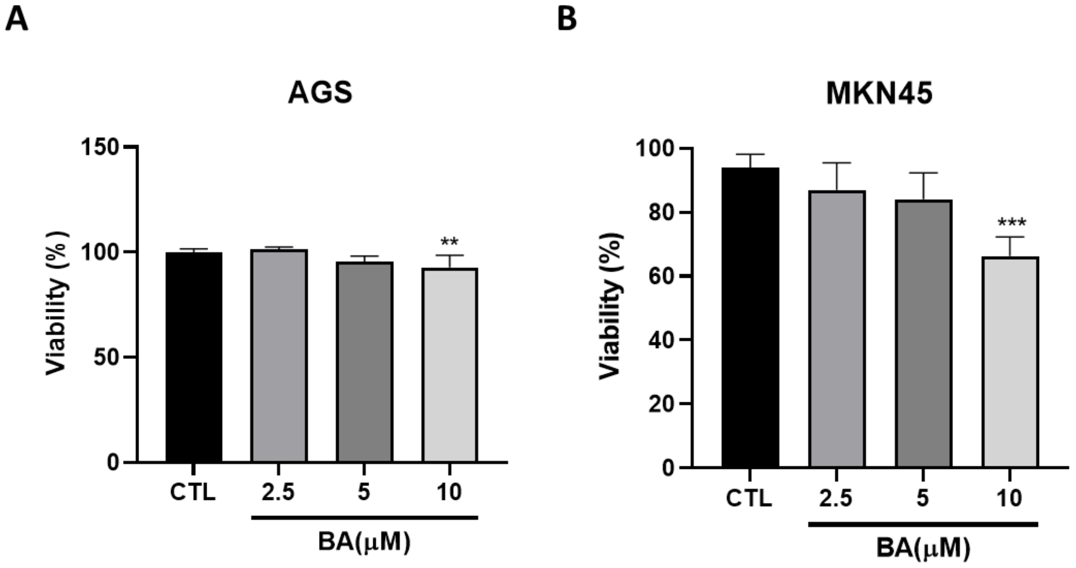

Figure S1. The human gastric cancer cells, AGS or MKN45 were treated BA from 2.5-10 μM for 48 h. The cell viability was evaluated by CCK-8 assay. The results revealed that BA inhibited human gastric cancer growth at 10 μM. Data are presented as mean ± SEM for four independent experiments. Significance was calculated by one-way ANOVA analysis: ***P* < 0.01.

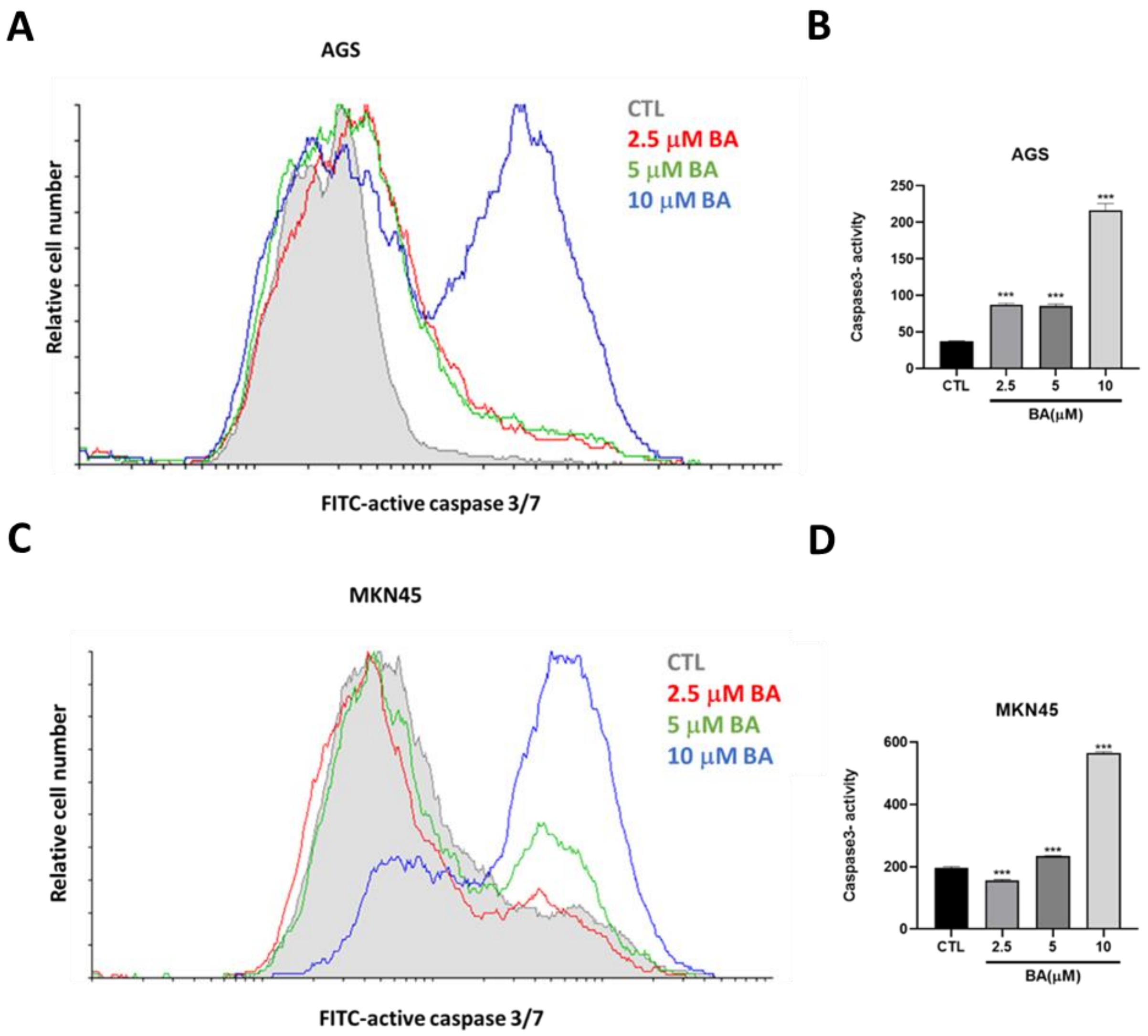

Figure S2. The human gastric cancer cells, AGS or MKN45 were treated BA from 2.5-10 μM for 48 h. The active caspase 3/7 was evaluated by using FITC Active Caspase-3 Apoptosis Kit. The results demonstrated that BA induced caspase 3/7 activation in human gastric cancer cells. Data are presented as mean ± SEM for four independent experiments. Significance was calculated by one-way ANOVA analysis: ***$P < 0.005$.

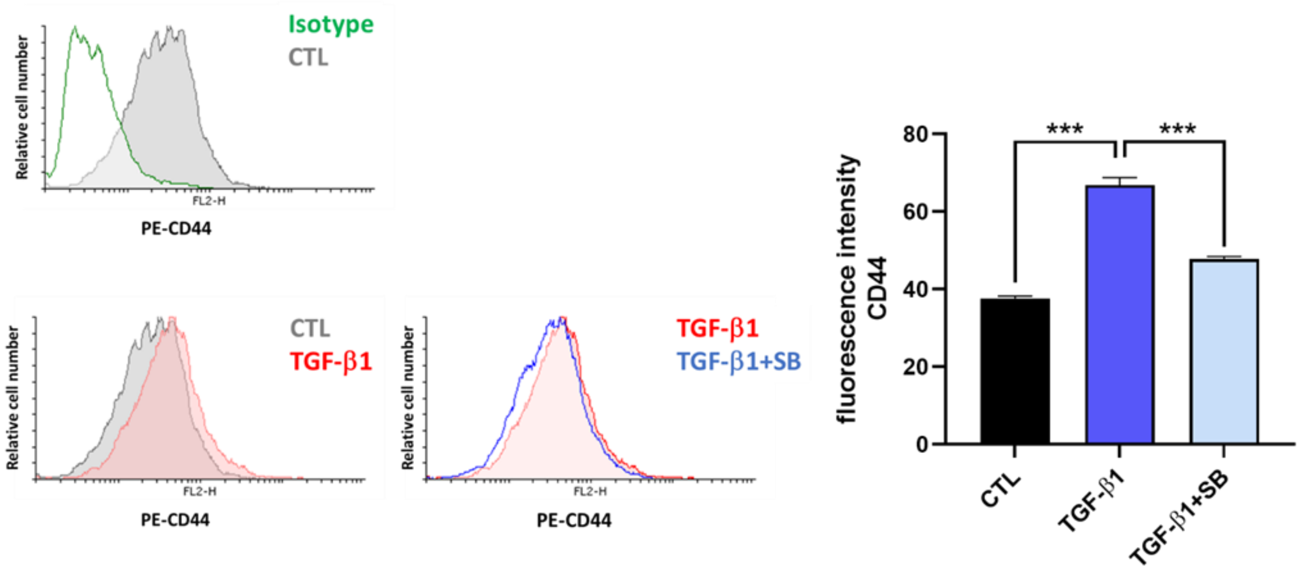

Figure S3. Surface marker CD44 histogram analyzed by flow cytometry after treatment with 20 ng/mL TGF-β1 and 10 μM SB431542 (TGF-β receptor inhibitor) for 48 h. Data are presented as mean ± standard error of the mean (SEM) for four independent experiments. Significance was calculated by one-way ANOVA analysis: ***$P < 0.005$.

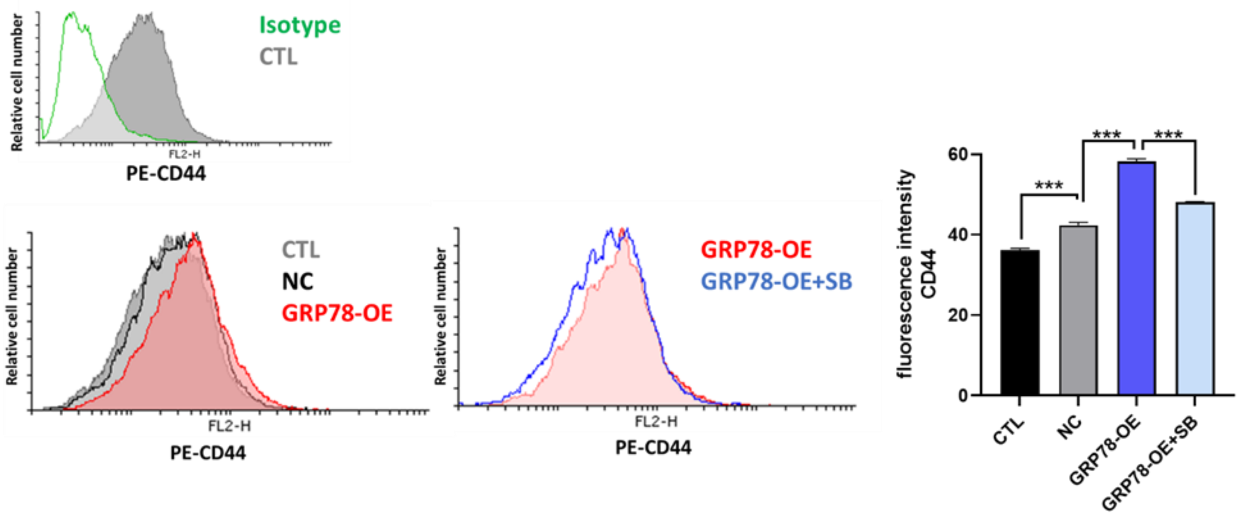

Figure S4. Surface marker CD44 histogram analyzed by flow cytometry in AGS after conditioned medium treatment for 48 h. Data are presented as mean ± standard error of the mean (SEM) for four independent experiments. Significance was calculated by one-way ANOVA analysis: ***$P <$ 0.005.

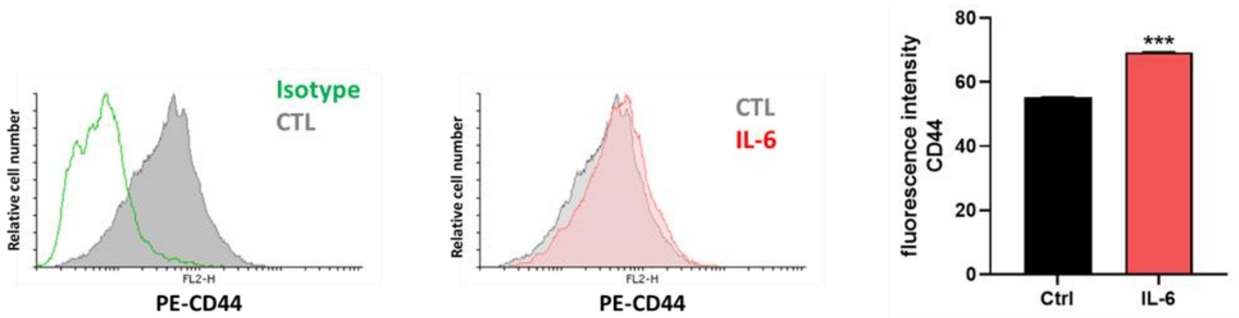

Figure S5. Surface marker CD44 histogram analyzed by flow cytometry in AGS after treatment with 20 ng/mL IL-6 for 48 h. Data are presented as mean ± standard error of the mean (SEM) for four independent experiments. Significance was calculated by one-way ANOVA analysis: ***$P < 0.005$.

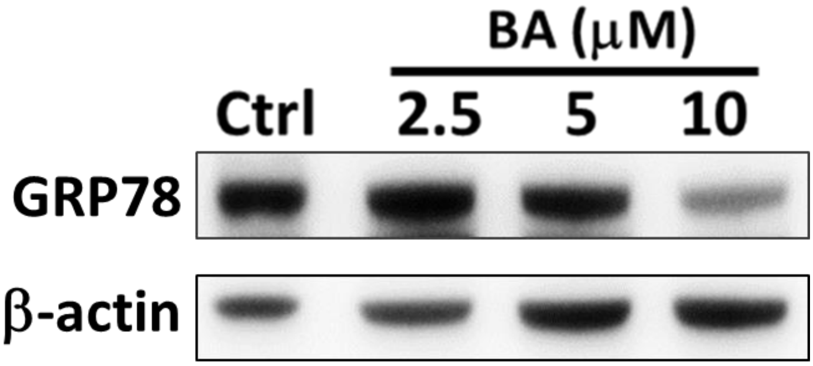

Figure S6. GRP78 expression by Western blotting analysis. Human gastric cancer MKN45 cells were treated with 2.5, 5, and 10 μM BA for 48 h. BA at 2.5 μM did not inhibit GRP78 within 48 h in MKN45. However, BA inhibited GRP78 from the dosage of 2.5 to 10 μM in AGS.

Cell viability analysis

The cells were seeded in 96-well plates in quadruplicate at 6000 cells/well and cultured for 24 hours before treatment. Cell viability was analyzed using the Cell Counting Kit-8 (CCK-8; Sigma-Aldrich, St. Louis, MO, USA) according to the manufacturer's instructions, and absorbance was measured at 450 nm by using a microplate reader.

Flow cytometry analysis

After treatment, the cells were washed with cold PBS and stained for 45 min with a surface marker antibody. After being stained, the cells were washed twice with cold PBS before analysis. Flow cytometry was then used to evaluate the expression of cancer stemness-related marker (CD44; BD Biosciences, San Jose, CA, USA) or active-caspase 3/7 (FITC-Active Caspase-3/7 Staining Kit) on human gastric cancer cells.

Western blot analysis

Human gastric cancer cells were washed with phosphate-buffered saline (PBS). Total protein samples were then extracted, and protein concentrations were measured using the Bradford protein assay (Bio-Rad, Hercules, CA, USA). Equal quantities of total proteins were separated using Bolt Bis-Tris Plus 4%–12% sodium dodecyl sulfate-polyacrylamide gel electrophoresis (Thermo Fisher Scientific, New York, NY, USA) and transferred onto polyvinylidene fluoride membranes. After the membranes were washed with PBS and Tween 20, they were then blocked with a blocking buffer (Bio-Rad) for 30 min at room temperature and incubated with primary antibodies GRP78 (1:1000; Cell Signaling Technology, Danvers, MA, USA) at 4°C. Finally, the membranes were incubated with secondary antibodies at room temperature for 1 h and analyzed using an electrochemiluminescence detection system.
